# Consolidation of cognitive maps as a gradual process

**DOI:** 10.1007/s00221-026-07236-7

**Published:** 2026-05-08

**Authors:** Otmar Bock

**Affiliations:** Institute of Exercise Training and Sport Informatics, German Sport University Cologne, Cologne, Germany

**Keywords:** Spatial cognition, Navigation, Consolidation, Interference

## Abstract

Cognitive maps of the environment are initially encoded in a fragile form susceptible to interference, but they can stabilize over time—a process called “consolidation”. This study investigated whether consolidation is a gradual or an all-or-none process. In the main condition, participants formed first a map of environment A, then a map of environment B, and then returned to A (sequence: A_1_ -> B -> A_2_). Performance increased from A_1_ to A_2_, but this increase was smaller than in a control condition where B was replaced by a pause filled with unrelated activities (sequence: A_1_ -> pause -> A_2_). Adding a pause after A_1_ in the main condition (sequence: A_1_ -> pause -> B -> A_2_) had no substantiable effect on performance. In contrast, adding a replica of A_1_ (sequence: A_1_ -> A_R_ -> B -> A_2_) improved performance so that it no longer differed significantly from the control condition. This pattern of findings is consistent with the view that (1) cognitive maps consolidate gradually rather than abruptly, and (2) consolidation proceeds during task learning but not during pauses filled with other activities.

## Introduction

Wayfinding through buildings, cities and landscapes often relies on internal representations of the environment, called “cognitive maps” (Tolman 1948). Such maps allow us to reach a variety of destinations from different starting points and along different routes. For example, we can travel along the shortest or the most scenic route, and plan a detour around a roadblock. This flexibility is not offered by alternative wayfinding strategies such as route following and approaching a beacon, which are limited to a fixed route or a single destination (Tlauka and Wilson 1994; Waller and Lippa 2007).

Cognitive maps are probably not exact models of the real world. They rather can be fragmented, distorted, influenced by assumptions, poor in detail, and sometimes even non-metric (Chrastil and Warren 2013; Peer et al. 2024). Depending on task demand, they can be anchored in different reference frames such as a person’s body (head, trunk or limbs), environmental boundaries, or cardinal directions (Galati et al. 2010; Meilinger et al. 2015; Richardson et al. 1999). Furthermore, they seem to represent multilevel buildings as a volume or as a stack of horizontal planes, depending on architecture (Bock 2025b; Jeffery et al. 2013; Lu and Ye 2019). It has further been reported that the fidelity of cognitive maps may not depend on the number of encoded locations, indicating that spatial information may be encoded in a holistic fashion (Bock 2025a).

It is well established from clinical and neurophysiological research that cognitive maps are formed in the hippocampus (Fritch et al. 2021; Nadel and Moscovitch 1997; O’Keefe and Nadel 1978). There, they are initially encoded in a fragile form that is susceptible to interference from new information. Over time, however, these representations are stabilized through increases in synaptic efficiency and thus become resistant to interference. This resistance to interference is referred to as “cellular consolidation”, in contrast to “systems consolidation” (Nadel and Moscovitch 1997). The latter involves memory transfer from hippocampal to neocortical circuits where it is stored in a less detailed, more generalized form. For reasons of brevity, “cellular consolidation” will henceforth be referred to as “consolidation”.

Consolidation has been examined at the behavioral level by asking participants to acquire spatial information A, then a different spatial information B, and finally to return to A (i.e., A_1_ -> B -> A_2_). In a control condition, acquisition of B was replaced by a pause of equal length, filled by a non-spatial task, everyday activities, or attentive rest). In this work, A_2_ performance was poorer in the main condition than in the control condition when B was administered within minutes after A_1_ but not when it was administered hours later (Darling et al. 2007; Eggert et al. 2014; Tresch et al. 1993; Zimmer et al. 2003). Thus, spatial memory formed during A_1_ became resistant to interference from B within less than a few hours. Since resistance to interference is widely regarded as a hallmark of consolidation (McGaugh 2000; Nadel and Moscovitch 1997), these findings were interpreted as behavioral evidence that consolidation of spatial memory can be achieved within less than a few hours.

The above work left open whether the interference observed with short delays between A_1_ and B was complete or partial. According to one view, vulnerable memory traces are “overwritten” by new information (Nairne 1990), which corresponds to complete erasure during Block B. According to another view, however, vulnerable memory traces “compete” with new information (Bancroft et al. 2016), which is consistent with partial degradation rather than complete erasure during Block B. Both views predict poorer A_2_ performance in the main compared to the control condition, and the aforementioned studies therefore cannot distinguish between them. To allow such a distinction, the present work evaluated not only A_2_ performance; it also quantified the improvement from A_1_ to A_2_. This allowed a distinction between the following possibilities:

- *No consolidation, hence complete interference* If memory traces formed during A_1_ are completely overwritten during B, then performance during A_2_ should follow the same trajectory as that during A_1_. Hence, the improvement (A_2_–A_1_) should be negligible in size.
- *Partial consolidation, hence partial interference* If the original memory traces are only partly degraded by competition during B, then A_2_ performance should benefit to some degree from knowledge acquired during A_1_. The improvement (A_2_–A_1_) should therefore be reliably larger than zero, yet smaller than that in the control condition.
- *Full consolidation, hence no interference* If the original memory traces are unaffected by B, then the improvement (A_2_–A_1_) should be comparable in size to that in the control condition.

The spatial tasks A and B adopted for the present work required participants to form a cognitive map of twelve objects encountered during six trips through a virtual maze. One set of objects was used by Task A, and a different set by Task B. Earlier work of the author’s group (e.g., Bock et al. 2024) indicates that these tasks take about ten minutes to complete and are neither too easy nor overwhelmingly difficult. Therefore, the working hypothesis was that these tasks would yield partial consolidation during A_1_, and consequently only partial interference by B. According to a supplementary hypothesis, extending the interval between A_1_ and B would allow consolidation to progress further and thus would reduce interference. The latter hypothesis was examined by introducing either a ten-minute pause filled with everyday activities or a ten-minute repetition of task A (= Block A_R_) before Block B was administered. Thus, supplementary condition 1 followed the sequence A_1_ -> pause -> B -> A_2_, and supplementary condition 2 followed A_1_ -> A_R_ -> B -> A_2_. The former condition addresses additional “offline” consolidation while attention is directed elsewhere, whereas the latter examines additional “online” consolidation through continued engagement with the target task.

If, as hypothesized, consolidation indeed is incomplete in the main condition and gains additional strength in the supplementary conditions, this would suggest that consolidation is a gradual process, evolving over time.

## Methods

### Participants

One hundred and twelve persons were recruited via the internet platform Prolific, with the inclusion criteria of age range 20–40 and fluency in English (since instructions were presented in English). Among them, 38 were assigned to the main condition (mean age 30.2 ± 9.2 years, 13 females), 38 to the control condition (mean age 29.4 ± 7.9 years, 14 females), 18 to supplementary condition 1 (37.1 ± 11.1 years of age, 9 females) and 18 to supplementary condition 2 (39.0 ± 15.6 years of age, 7 females). None of the participants had taken part in the author’s previous wayfinding studies. Because the present experiments were conducted online, all participants were necessarily computer-literate. On average, therefore, they may have had more experience with video games that involve spatial skills than the general population. At the same time, they likely had less real-world navigation experience than population members whose work depends heavily on spatial orientation, such as delivery drivers or field technicians.

The study was part of a larger research program pre-approved by the author’s institutional Ethics Commission (Approval No. 062/2020). Informed consent was obtained from each participant before testing.

### Cognitive mapping task

**Fig. 1:**
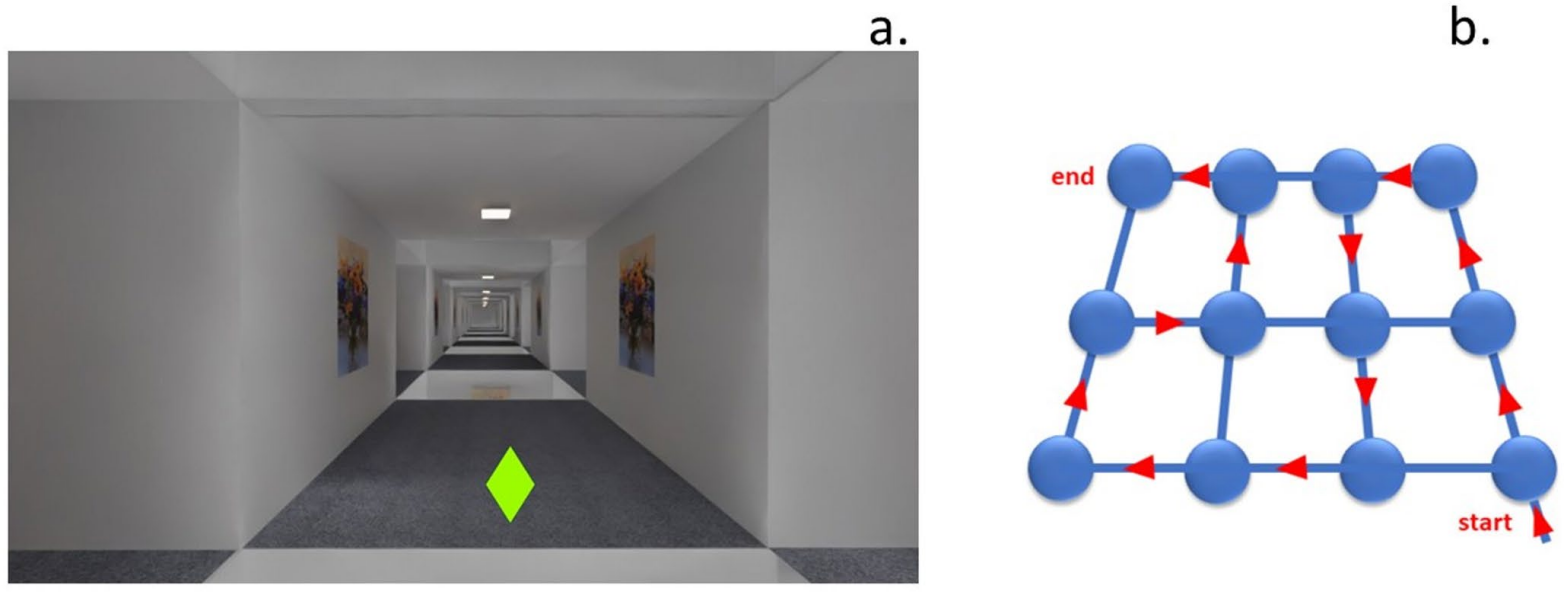
**a** Exemplary screenshot of a maze intersection as seen by participants on learning trips. The green diamond is the shape presented at this particular intersection. **b** Schematic drawing of the maze, with blue discs symbolizing intersections and blue lines the connecting corridors. This drawing was shown to participants before each learning trip, with red arrows indicating the upcoming route, and was shown during the test without arrows

The task was administered remotely on the participants’ desktop computers via the online platform Gorilla. To ensure consistency with earlier work of the author’s group, cognitive mapping was assessed using virtual grid mazes with a plain and uniform interior design. A unique geometric shape was placed at each of twelve adjacent grid intersections (see Fig. 1b). During ***learning trips***, participants had to memorize the locations of these shapes; during interleaved ***tests***, they had to indicate the location of each shape on a schematic drawing of the maze. Abstract geometric shapes, rather than realistic objects, were used to minimize encoding by narratives (e.g., “the bulldozer drove through the tent to hit the tower”).

During each ***learning trip***, participants viewed a virtual tour through the maze from a first-person perspective (as in Fig. 1a). They travelled from one intersection to the next within 2 s, and then stopped for 2.5 s while the geometric shape located at that intersection was displayed straight ahead. The trips included no left or right turns. For example, instead of a right turn participants saw a sideways shift, as if looking through the side window of a moving vehicle. Instead of a subsequent right turn they saw a backward shift, as if looking through the vehicle’s rear window. In this way, participants’ egocentric straight-ahead was aligned with the mazes’ allocentric North throughout the trip. Such an alignment facilitates spatial orientation in a virtual grid maze (Bock 2025b), likely by avoiding the cognitive demand associated with rotational transformations between egocentric and allocentric reference frames.

Each ***test*** displayed a schematic drawing of the maze (see Fig. 1b, but without red arrows), together with one of the previously encountered geometric shapes. Participants indicated the shape’s location by clicking on the corresponding intersection with the mouse. When the response was correct, the next shape appeared immediately. When the response was incorrect or not given within 12 s, an error message was displayed for 3 s before the next shape appeared, etc., until all twelve shapes had been responded to. This testing procedure avoids the response bias from inaccurate self-orientation that would arise if participants were tested by asking them to re-visit the shapes in first-person perspective (Bock 2025a).

### Procedures

Participants were informed that they would proceed through virtual mazes and subsequently report the locations of geometric shapes encountered along the way. For familiarization, they first completed a short learning trip forward across three intersections, followed by a test. Unlike in the main experiment, this test required each incorrect response to be repeated until a correct response was given. Familiarization then continued with a second learning trip, this time rightward across three intersections, and a second test. Thus, by the end of familiarization, each participant had given six correct responses.

The actual experiment began with Block A_1_, the first block of trials in which participants acquired cognitive map A. In this block, learning trips (L) and tests (T) alternated according to the sequence L-L-T-L-T-L-T-L-T-L-T. Each learning trip took a different route through the maze. Therefore, participants encountered the same shapes at the same intersections—but in a different serial order. The serial order of shapes also differed between tests, so that each learning trip and each test used a different order. This variation was implemented to prevent reliance on serial order as a mnemonic cue. At the onset of each learning trip, participants were shown a schematic drawing of the maze, but with arrows indicating the upcoming route (see example in Fig. 1b). This was to facilitate self-orientation during the trip. At the same time, participants were reminded of their task to memorize the locations rather the serial order of shapes.

Depending on the condition to which participants were assigned, Block A_1_ was followed by

- Block B, in which participants acquired a different cognitive map,
- a pause, in which participants engaged in everyday activities (compliance with this instruction was confirmed during debriefing), or
- Block A_R_, which was a replica of Block A_1_.

The experiment ended for all conditions with Block A_2_, another replica of A_1_. The order of blocks was A_1_ -> B -> A_2_ in the main condition, A_1_ -> pause -> A_2_ in the control condition, A_1_ -> pause -> B -> A_2_ in supplementary condition 1, and A_1_ -> A_R_ -> B -> A_2_ in supplementary condition 2. The duration of each block was about 10 min. Intervals between blocks were a few seconds, depending on the time participants needed to read the instruction screen.

The virtual mazes used in Block A and B differed only in that the color and location of shapes was not the same. For instance, one maze displayed a red triangle at the front-far-left intersection, whereas the other displayed a purple triangle at the rear-near-left intersection. The assignment of mazes to Blocks A and B was balanced across participants from each condition.

### Data analysis

Performance was quantified as response accuracy, defined as the proportion of correct responses on each test. Since this metric was bounded by 0 and 1 and therefore was not normally distributed, statistical analysis took a logistic approach: a generalized linear mixed-effects model with binomial error distribution and logit link function. The model included the fixed effects Condition, Block (A_1_, A_2_), Test (1 to 5), all their interactions, and Sex (f, m). A random intercept for participant ID accounted for repeated measures. The model was fitted using the R function *glmer* from the lme4 package, using the *bobyqa* optimizer to ensure convergence.

To examine the primary hypothesis (attenuated consolidation in the main compared to the control condition) the two planned comparisons described in the Introduction section were carried out. The levels of fixed effect Condition were set to (main, control), and the model outcome was analyzed with the R functions *emmeans* and *contrast* from the emmeans package to compute two planned contrasts. One contrast compared A_2_ versus A_1_ within the main condition, and the second compared the performance improvement (A_2_–A_1_) in the main versus in the control condition. Statistical significance was assessed on the model’s logit scale without correcting for multiple-comparisons, consistent with the a priori specification of these contrasts. Effect sizes were computed following Chinn (2000), by multiplying each logit coefficient by √3/π to obtain a metric comparable to Cohen’s *d* under logistic regression. Benchmarks for small, medium, and large effects are d = 0.2, 0.5, and 0.8, respectively (Cohen 1988).

To evaluate the supplementary hypothesis (more consolidation by an additional pause or additional learning), two models were fitted for each supplementary condition. Condition levels were (supplementary, control) in one model, and were (supplementary, main) in the other.

Required sample size n_r_ was estimated with G*Power (Faul et al. 2007), using f = 0.25, r = 0.5, a = 0.05 and ß = 0.95, yielding n_r_ = 54 both for a within-condition comparison of two repeated measures (contrast 1) and for a within-between comparison of two conditions x two repeated measures (contrast 2). Hence the actual sample sizes, n = 76 for the main hypothesis and n = 56 for the supplementary hypothesis, were larger than n_r_.

## Results

**Fig. 2:**
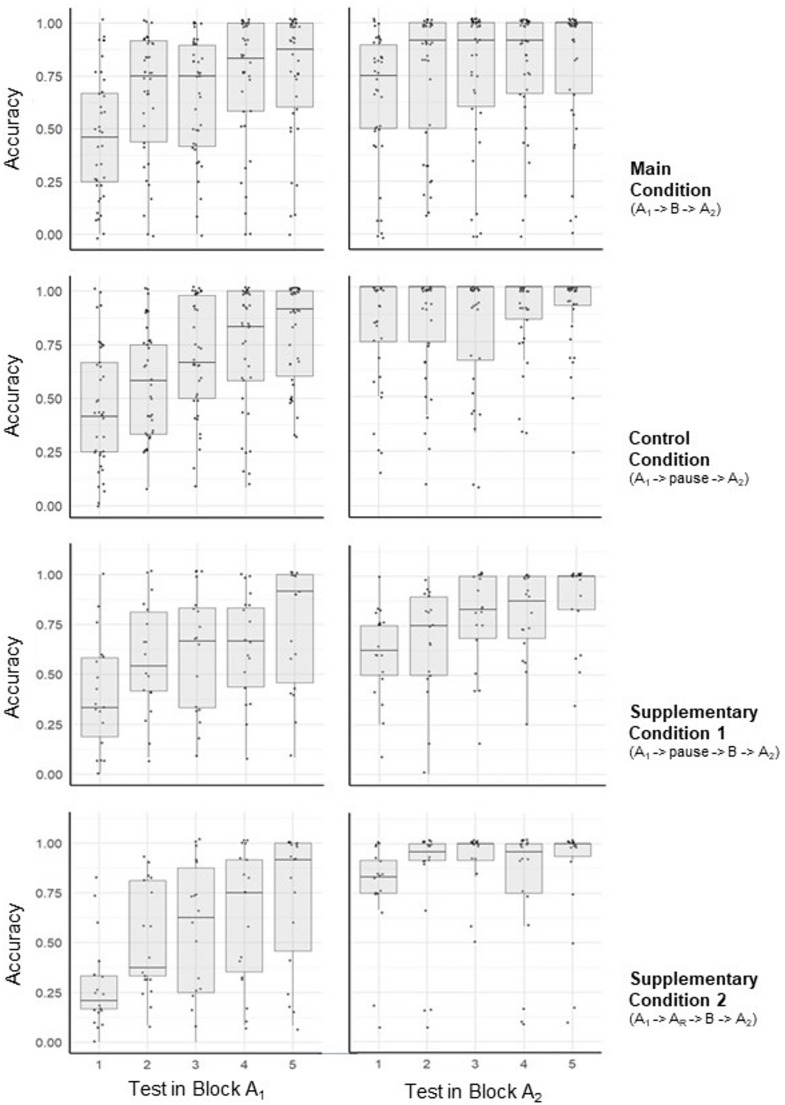
Accuracy in all conditions and tests in Block A_1_ (left) and A_2_ (right). Each dot represents the accuracy of one person in one test; the dots are jittered horizontally and vertically to reduce overlap. By definition, accuracy is bounded by 0 and 1. Tukey style box plots are overlaid to visualize data distributions

**Fig. 3:**
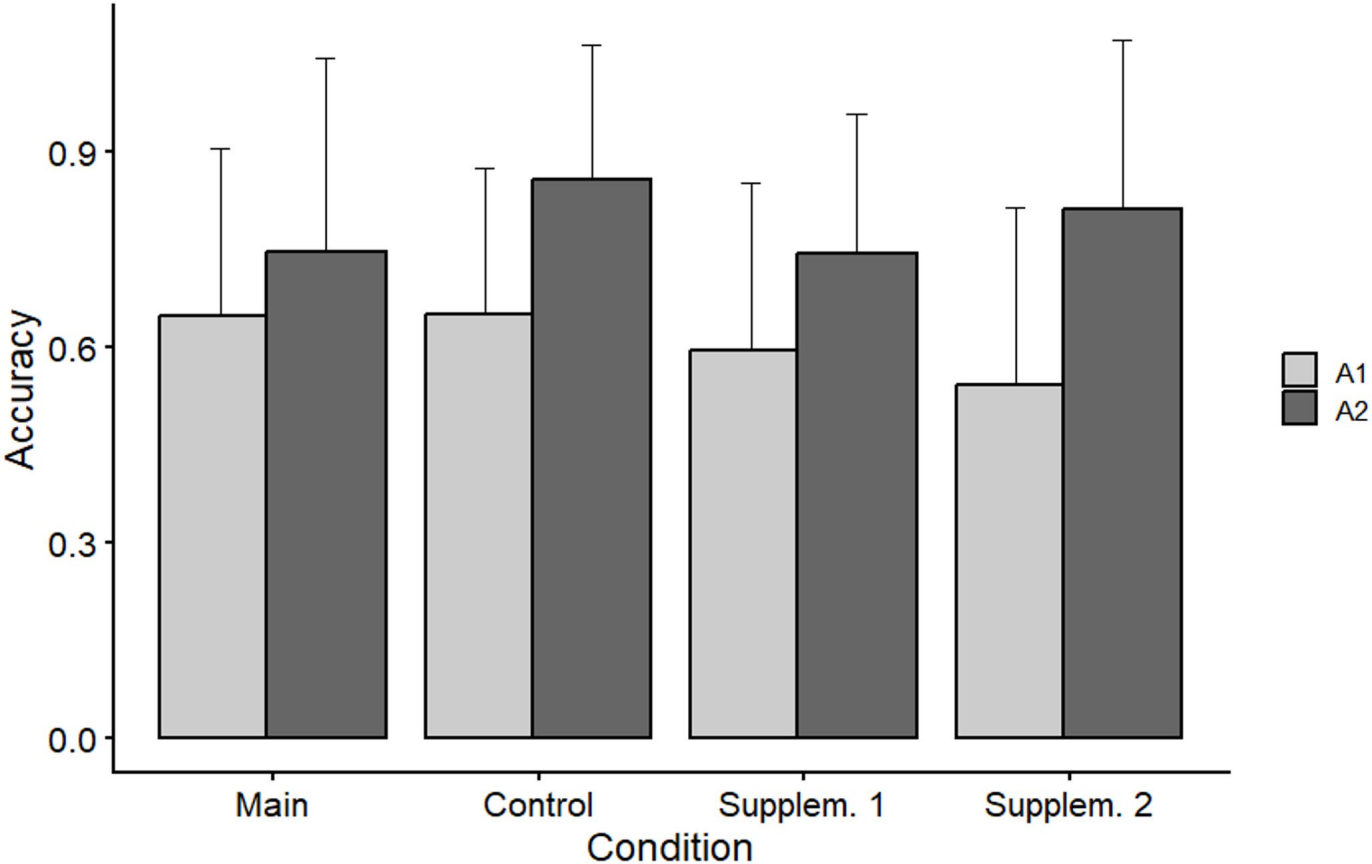
Accuracy in Block A_1_ and A_2_ of all conditions. Bars are means across participants and tests, and error indicators are the pertinent standard deviations. Note that unlike in the statistical analyses, these data are not corrected for the effects of Sex, Test, and interactions including Sex and/or Test

Figure 2 shows the data registered in the four conditions during Block A_1_ and A_2_. Response accuracy varied widely, as known from earlier work by our group and others. Nonetheless, there is a distinct trend for accuracy to increase from test to test in Block A_1_, and to be generally higher in Block A_2_ compared to A_1_. Figure 3 summarizes these data across participants and tests.

Table 1 exemplifies the outcome of omnibus statistics when Condition was set to (main, control). Significant and substantial effects emerged for Block, Test, and all interactions—a pattern that is difficult to interpret. The present analysis therefore focused on the hypothesis-driven planned comparisons described in Introduction and Methods.

**Table 1:** Results of Type-III likelihood-ratio tests and the corresponding effect sizes^a^

Effect	χ^2^	df	*p*	R^2^
Sex	0.049	1	0.824	< 0.001
Condition	2.298	1	0.130	0.003
Block	343.199	1	** < 0.001**	**0.410**
Test	366.235	4	** < 0.001**	**0.383**
Condition x Block	48.262	1	** < 0.001**	**0.058**
Condition x Test	11.240	4	**0.024**	**0.013**
Block x Test	42.808	4	** < 0.001**	**0.051**
Condition x Block x Test	12.882	4	**0.012**	**0.015**

^a^ Tests were computed in the R function *Anova* from the car package, Effect sizes R^2^ were computed as χ^2^_effect / Σχ^2^_total), as originally proposed by Nagelkerke (1991) for generalized linear models. Benchmarks for small, medium and large effects are R^2^ = 0.01, 0.06, and 0.14, respectively (Cohen 1988). Significant effects are highlighted in bold.

When Condition levels were set to (main, control), the contrast comparing A_2_ versus A_1_ within the main condition was significant (z = 6.947, *p* < 0.001, d = 0.398). Likewise, the contrast comparing the improvement (A_2_–A_1_) in the main versus in the control condition was significant (z = 8.622, *p* < 0.001, d = 0.390). Hence performance in the main condition improved from A_1_ to A_2_, but this improvement was smaller than in the control condition.

With Condition levels set to (supplementary 1, control), the within-condition contrast was again significant (z = 8.293, *p* < 0.001, d = 0.497), as was the between-condition contrast (z = 4.751, *p* < 0.001, d = 0.372). However, when Condition levels were set to (supplementary 1, main), the between-condition contrast was not significant (z = 1.430, *p* = 0.153, d = 0.108). Hence the data do not support the view that improvement from A_1_ to A_2_ differed between supplementary condition 1 and the main condition.

With Condition levels set to (supplementary 2, control), the within-condition contrast was significant (z = 14.416, *p* < 0.001, d = 0.961) but the between-condition contrast was not (z = 1.085, *p* = 0.228, d = 0.091). When Condition levels were set to (supplementary 2, main), the between-condition contrast became significant (z = 10.934, *p* < 0.001, d = 0.981). Hence there is no support for the view that improvement in supplementary condition 2 differed from that in the control condition.

To explore whether low-performing participants disproportionately influenced the above results, the analysis was repeated after excluding individuals with mean A_1_ accuracy below 0.333. These were five participants from the main condition, one from the control condition, three from supplementary condition 1 and five from supplementary condition 2. Table 2 shows that the significance pattern did not change, indicating that findings were not unduly driven by poor performers.

**Table 2:** Outcome of planned contrasts within and between conditions^a^

Levels of condition	Within-condition contrast	Between-conditions contrast
Main, control	**z = 9.406, *p* < 0.001, d = 0.475**	**z = 5.312, *p* < 0.001, d = 0.383**
Supplementary 1, control	**z = 6.972, *p* < 0.001, d = 0.455**	**z = 4.850, *p* < 0.001, d = 0.404**
Supplementary 1, main	“	z = 0.236, *p* = 0.813, d = 0.019
Supplementary 2, control	**z = 10.949, *p* < 0.001, d = 0.983**	z = 1.200, *p* = 0.230, d = 0.124
Supplementary 2, main	“	**z = 10.934, *p* < 0.001, d = 0.981**

^a^ Significance is highlighted in bold.

## Discussion

The present study examined the consolidation of cognitive maps. In accordance with established theory (McGaugh 2000; Nadel and Moscovitch 1997), consolidation was operationalized as resistance to interference by another cognitive map. Thus, full consolidation would be indicated by no interference, partial consolidation by partial interference, and no consolidation by complete interference.

The primary hypothesis was that consolidation is a gradual process, such that if the task is neither too easy nor too difficult, one should observe partial interference. To find out, performance in the main condition (A_1_ -> B -> A_2_) was compared to a control condition (A_1_ -> pause- > A_2_). A planned contrast revealed that the improvement (A_2_–A_1_) was smaller in the main condition than in the control condition, consistent with earlier evidence that recently acquired spatial memory is vulnerable to interference from subsequent spatial learning (Darling et al. 2007; Tresch et al. 1993; Zimmer et al. 2003). Importantly, however, another planned contrast extended this earlier work: the improvement in the main condition, although smaller, was nevertheless of substantial size. This indicates that spatial information acquired during A_1_ partly resisted interference and thus contributed to A_2_ performance. This improvement is not an artifact of additional learning during A_2_: if interference had completely abolished the information acquired during A_1_, then A_2_ learning would have followed the same trajectory as A_1_ learning, with no noticeable improvement. Nor is the improvement likely an artifact of task-unspecific practice (e.g., better understanding of methods and requirements, procedural fluency, strategic adjustments): if such task-unspecific benefits had continued to grow beyond the six familiarization trials and into Block A_1_, then not only A_2_ performance but also B performance should have exceeded A_1_ performance—yet this was not observed (across-participant mean accuracy ± SD in the main condition: A_1_ = 0.649 ± 0.287; B = 0.647 ± 0.259; hence no unspecific practice benefits for B). In sum, the robust but smaller improvement in the main compared to the control condition indicates that the map formed during A_1_ was partly deleted during B but partly resisted interference. This is consistent with partial consolidation as predicted by the primary hypothesis.

The supplementary hypothesis proposed that a longer interval between A_1_ and B would allow consolidation to progress further and thus reduce interference. Planned contrasts showed that extending the interval by a ten-minute pause (supplementary condition 1: A_1_ -> pause -> B -> A_2_) resulted in an improvement (A_2_–A_1_) comparable to the main condition. Thus, the partial interference observed in the main condition was not noticeably reduced by a pause, indicating that consolidation did not continue any further during the pause. In contrast, extending the interval by an additional ten minutes of first-map learning (supplementary condition 2: A_1_ -> A_R_ -> B -> A_2_) yielded an improvement (A_2_–A_1_) comparable to the control rather than to the main condition. Hence, the partial interference observed in the main condition was no longer detectable when the opportunity for learning the first cognitive map was extended, indicating that the first map was fully consolidated. This outcome supports a qualified version of the supplementary hypothesis. Thus, consolidation was apparently completed within ten extra minutes of task learning, but progressed little within ten extra minutes of pausing. Continued task engagement therefore seems to be critical for continued consolidation in the present study.

The differential effect of a pause versus continued learning may relate to earlier work showing that spatial memory deteriorates when attention is engaged elsewhere (Awh et al. 1998; Craig et al. 2016). By analogy, the consolidation process may also deteriorate when it is not in the focus of attention. This could explain why a pause filled with everyday activities, which likely diverted participants’ attention, did not allow consolidation to progress, whereas additional task learning, which likely maintained attention on the relevant memory traces, did allow continued consolidation. If this interpretation is correct, then replacing the pause with a period of wakeful rest should also support consolidation, since attention would also not be distracted by other activities (Craig et al. 2016). This prediction should not be explored by online experiments such as the present ones; rather, an experimenter should be on site to ensure participants’ adherence to the instructed period of wakeful rest.

**Fig. 4:**
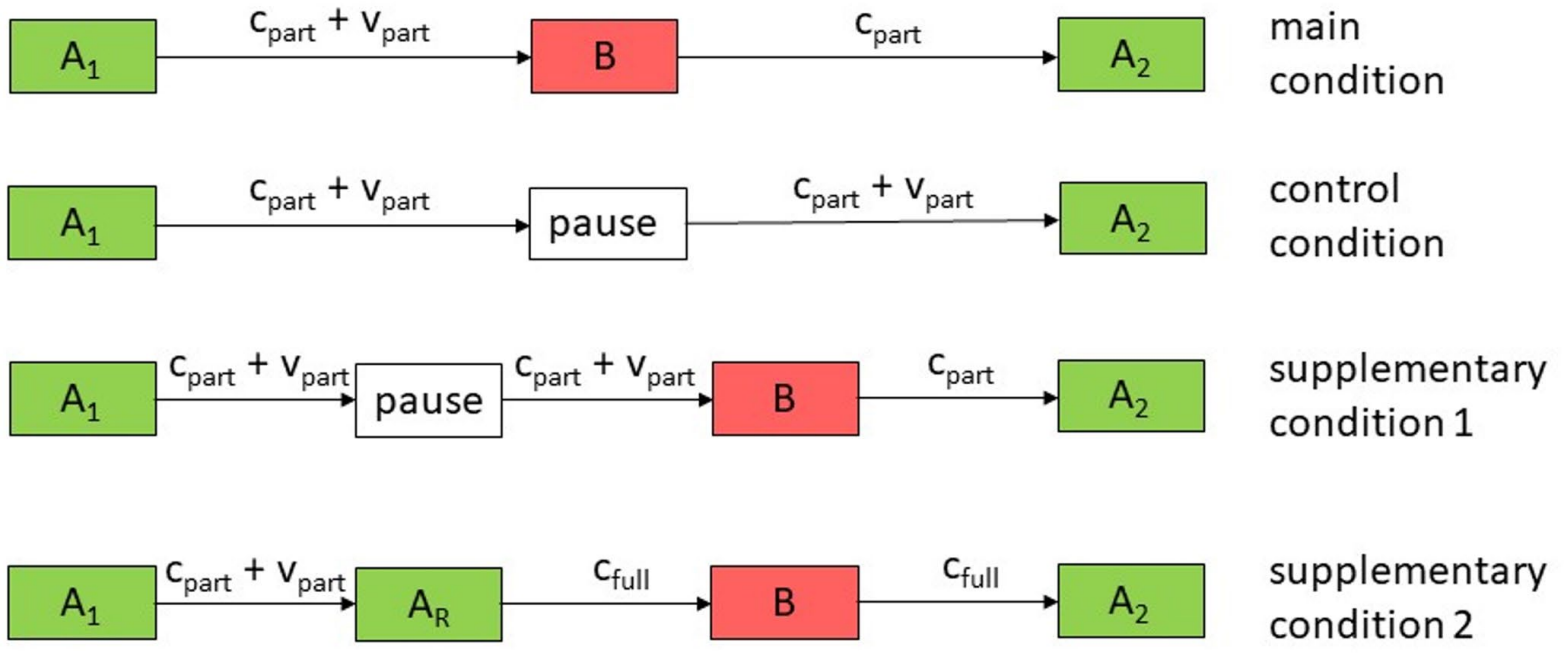
Schematic representation of possible consolidation processes in the present study. c_part_ + v_part_ refer to partial consolidation of the first map combined with partial vulnerability to interference, and c_full_ to strong or complete consolidation of the first map with little vulnerability to interference. Arrows represent the flow of spatial information but not the passage of time, as all blocks were scheduled in immediate succession

Taken together, the present findings support a conceptual framework according to which cognitive maps consolidate gradually through learning. Partial consolidation seems to be achieved within ten minutes of learning, and full consolidation within additional ten minutes of learning but not within ten additional minutes of unrelated activities. Figure 4 illustrates how this framework could explain the present pattern of findings:

- During Block A_1_ in all conditions, the first cognitive map partly consolidated and partly remained vulnerable to interference (c_part_ and v_part_ in Fig. 4, respectively).
- In the main condition, v_part_ was degraded during Block B while c_part_ persisted. In the control condition, however, v_part_ was largely preserved during the pause so that both v_part_ and c_part_ persisted. In the main condition, therefore, A_2_ performance exceeded A_1_ performance but the (A_2_–A_1_) difference was smaller than in the control condition.
- In supplementary condition 1, v_part_ was also largely preserved during the pause but was degraded during the subsequent Block B, so that only c_part_ persisted. Therefore, A_2_ performance exceeded A_1_ performance but the (A_2_–A_1_) difference was again smaller than in the control condition and resembled that in the main condition.
- In supplementary condition 2, consolidation of the first map proceeded during A_R_ (c_full_ in Fig. 4), so that the first map became largely resistant against interference during Block B. Consequently, the (A_2_–A_1_) difference resembled that in the control condition and was larger than in the main condition.

Future research should refine this conceptual framework in several ways. Rather than contrasting one versus two blocks of task learning, the amount of learning should be varied gradually to determine the quantitative relationship between learning and consolidation. Furthermore, activities during pauses could be varied to clarify the conditions under which interference arises—whether it is limited specifically to the formation of another map, or extends to mental rotation and other tasks involving visuo-spatial processing. Studies could also examine whether the relationship between learning and consolidation depends on individual differences known to influence spatial cognition, such as sex, age, anxiety or risk-taking propensity (Hegarty et al. 2022).

The proposed conceptual framework may not necessarily generalize to other experimental paradigms or to real-world situations. First, spatial environments differ in many respects such as spatial layout (ranging from regular grids to a mix of X-, Y-, T- and star-shaped junctions), visual diversity (ranging from monotonous sceneries to those with many contrasting elements), and realism (ranging from schematic to photorealistic). Second, self-motion cues can be provided by optic flow but not locomotion (as in the present work), by optic flow and locomotion (as typical in real life), or by neither of these (when participants view static images on a desktop screen). Third, the computational demand for transforming between egocentric and allocentric reference frames can be high (first-person trips with left and right turns), lower (first-person trips without turns) or lower still (participants study physical cartographic maps). These and other factors may influence how interfering tasks and everyday activities affect the formation, consolidation and degradation of cognitive maps. These potential determinants should be addressed by future research.

In conclusion, within the methodological constraints of the present study, cognitive maps appear to consolidate gradually during periods of learning, but less so during engagement in other activities. Consolidation may therefore not be as abrupt as the metaphor of memory “overwriting” seems to suggest.

## Data Availability

The code for running the experiments and the datasets generated and analyzed in the current study are available from the author on reasonable request.
